# Beliefs and perceptions about the causes of breast cancer: a case-control study

**DOI:** 10.1186/1756-0500-7-558

**Published:** 2014-08-21

**Authors:** Allyson K Thomson, Jane S Heyworth, Jennifer Girschik, Terry Slevin, Christobel Saunders, Lin Fritschi

**Affiliations:** School of Occupational Therapy and Social Work, Curtin University, GPO Box U1987, Perth, WA Australia 6845; School of Population Health, The University of Western Australia, 35 Stirling Highway, Crawley, WA Australia 6009; Western Australian Institute for Medical Research, The University of Western Australia, Ground Floor, B Block, QEII Medical Centre, Hospital Avenue, Nedlands, WA 6009 Australia; Cancer Council Western Australia, 15 Bedbrook Place, Shenton Park, WA Australia 6008; School of Surgery, The University of Western Australia, 35 Stirling Highway, Crawley, WA Australia 6009; School of Public Health, Curtin University, GPO Box U1987, Perth, WA Australia 6845

**Keywords:** Western Australia, Breast cancer, Causes, Beliefs, Case-control

## Abstract

**Background:**

Attributions of causality are common for many diseases, including breast cancer. The risk of developing breast cancer can be reduced by modifications to lifestyle and behaviours to minimise exposure to specific risk factors, such as obesity. However, these modifications will only occur if women believe that certain behaviours/lifestyle factors have an impact on the development of breast cancer.

**Method:**

The Breast Cancer, Environment and Employment Study is a case-control study of breast cancer conducted in Western Australia between 2009 and 2011. As part of the study 1109 women with breast cancer and 1633 women without the disease completed a Risk Perception Questionnaire in which they were asked in an open-ended question for specific cause/s to the development of breast cancer in themselves or in others. The study identified specific causal beliefs, and assessed differences in the beliefs between women with and without breast cancer.

**Results:**

The most common attributions in women without breast cancer were to familial or inherited factors (77.6%), followed by lifestyle factors, such as poor diet and smoking (47.1%), and environmental factors, such as food additives (45.4%). The most common attributions in women with breast cancer were to mental or emotional factors (46.3%), especially stress, followed by lifestyle factors (38.6%) and physiological factors (37.5%), particularly relating to hormonal history.

**Conclusions:**

While the majority of participants in this study provided one or more causal attributions for breast cancer, many of the reported risk factors do not correspond to those generally accepted by the scientific community. These misperceptions could be having a significant impact on the success of prevention and early detection programs that seek to minimise the pain and suffering caused by this disease. In particular, women who have no family history of the disease may not work to minimise their exposure to the modifiable risk factors.

## Background

Breast cancer is one of the most commonly diagnosed invasive cancers and it accounts for a quarter of the female cancers diagnosed annually in Australia [1]. In Western societies it is estimated that one in nine women will have breast cancer before the age of 85 [1]. Increasing age, reproductive factors, mammographic density and, in around 9% of cases, genetic factors and family history are all known risk factors for breast cancer [2, 3]. A range of factors, such as dietary fat intake, and solvent and pesticide exposure, have also been implicated as potential risk factors but the evidence so far has been inconclusive [4–6]. For most cases of breast cancer, the true cause remains unknown [6].

Studies worldwide have identified many factors to which women with breast cancer attribute their condition, including stress and other psychosocial factors; knocks, bruises or injury to the breast; religious causes; chemicals, food additives; proximity to electronic equipment or overhead power lines; viral or bacterial infection; and bad luck [7–9], although there is limited evidence to support the attributions [10, 11]. Women without breast cancer also frequently identify similar risk factors when asked about their beliefs about the causes of breast cancer [12].

This study aimed to investigate perceptions about breast cancer causation in a study of women participating in a case-control study of breast cancer. Specifically, we aimed to investigate whether women attributed specific cause/s to the development of breast cancer in themselves or in others; what these causal beliefs might be; and to explore whether there may be differences in beliefs between women with breast cancer and women without the disease.

Understanding how women attribute breast cancer causation is important information for health promotion, including breast awareness and promotion of screening programmes, clinical care and policy development. Causal attributions are known to affect the responses of women to health messages and their adoption of various behaviours aimed at risk reduction, including mammographic screening [13, 14]. A poor understanding of known risk factors would highlight a need for health promotion messages to improve the knowledge and awareness of true risk factors. This would allow women to make more informed choices in adopting behaviours that influence their risk of developing and successfully treating the disease.

This paper describes the beliefs regarding breast cancer causes held by women in Western Australia with and without the condition.

## Method

Data for this study were collected as part of the Breast Cancer, Environment and Employment Study (BCEES), a case-control study of breast cancer conducted in Western Australia between 2009 and 2011 [15–18]. Within BCEES 1205 women with breast cancer diagnosed within the previous six-months (case women) from the WA Cancer Registry, and 1789 age-group matched women selected randomly from the electoral roll (control women) were recruited.

### Recruitment procedure and data collection

#### Data collection

Consenting BCEES participants, 1205 with breast cancer (response fraction: 57.8%) and 1789 without breast cancer (response fraction: 41.1%), completed an initial questionnaire which comprised sections on demographics, reproductive history, family history of breast cancer, sleep habits, employment history, lifetime physical activity, lifestyle, and pesticide exposure. Information about breast cancer stage and treatment was not sought.

Of the eligible women who did not participate, those who refused were older compared with those who did not respond. Case women who did not respond were more likely to live in very remote areas (4% compared with 2% who refused). There were no differences in socio-economic status between women who participated and those who did not.

After completion and return of the initial questionnaire participants were mailed a Risk Perception Questionnaire (RPQ). The RPQ consisted of a series of possible exposures and the woman was asked if each exposure increased the risk of breast cancer, decreased it, had no effect or they didn’t know [paper submitted].

The RPQ also included one of two possible open-ended questions: case women were asked "What do you believe caused your breast cancer?" while control women were asked "What do you believe causes breast cancer?". We allowed space for approximately 8-10 sentences. The RPQ was completed and returned by 1109 (92.0%) of case participants and by 1633 (91.3%) of control participants.

Written responses from the RPQ were converted into digital text using Cardiff TeleForms® software and reviewed by project staff for accuracy and completeness.

As there were 37 separate possible exposures listed in the main section of the RPQ, this paper describes only the responses to the single open-ended question. The quantitative data are the focus of a separate paper currently under review.

#### Data analysis

The text data were analysed using NVivo 9 (QSR International, 1999-2011), a qualitative data management software package. The responses for each person were initially reviewed by a single investigator (AT), and words or phrases from the text were coded within a scheme based on concepts identified from a range of sources [8, 9, 14, 19–24], as a form of directed content analysis [25].

After the initial review, the BCEES study team reviewed the categories, some example responses and any uncategorised text in order to further define and refine the coding definitions. A total of 37 categories were identified (including a category for ‘don’t know’ responses), representing six major themes. Respondents who wrote that they didn’t know, but then listed a number of possible causes were coded for the specific factors rather than ‘Don’t know’.

The coding definitions were then validated by two independent raters coding a random sample of responses. Discrepancies between raters were discussed with the BCEES study team and consensus was sought.

Analysis was conducted on the six higher level categories representing major themes in the data. These are described below:

#### Physiological

Included breast density and size, reproductive and hormonal profile, injury, disease and infection, immune system imbalance, sleep disturbance, medications, age, gender, and diagnostic delay.

#### Environmental

Included environment, chemicals, food additives, radiation, domestic and personal care products, passive smoking, pesticides, and shiftwork.

#### Familial

Included family history and inheritable genetic mutations, and a universal gene or cell that could turn malignant.

#### Mental/emotional

Included stress, mental illness, and other psycho-social aspects.

#### Modifiable/lifestyle

Included alcohol, poor diet, overweight/obese, hormone replacement therapy, physical inactivity, smoking, substance abuse, and unhealthy lifestyle.

#### Chance

Included fate, religious causes and (bad) luck

The modifiable/lifestyle category comprised the factors which fall under personal control. All other higher-level categories were considered to contain non-modifiable factors.

In the results below, quotations in the respondent’s exact words are used to illustrate causal attributions. The examples were chosen to demonstrate themes and are not necessarily representative of all respondents. Some editing has been necessary due to the length of some replies.

Descriptive analyses were conducted using Stata 12 (Statacorp, College Station, TX, USA).

Ethics approval for this project was received from the Human Research Ethics Committees of The University of Western Australia and the Department of Health Western Australia. All participants provided written informed consent.

## Results

### Participants

Case women were slightly younger, more likely to have a family history, and were more likely to have been born outside Australia and to have completed a university education than control women (Table 1). More than three-quarters of the participants provided one or more breast cancer causes in response to the open ended question in the RPQ (Table 1). However, case women twice as often responded ‘Don’t know’ and gave, on average, fewer suggested causes for breast cancer (case mean 2.2, control mean 3.1, based on the 37 concepts originally coded).

**Table 1:** **Demographic data for BCEES Risk Perception Questionnaire respondents: cases (n = 1109) and controls (n = 1633)**

	Cases N (%)	Controls N (%)
Age at recruitment*		
49 years or less	311 (28.0)	337 (20.6)
50-59 years	303 (27.3)	485 (29.7)
60-69 years	321 (28.9)	554 (33.9)
70 years or older	174 (15.7)	257 (15.7)
**Country of birth**		
Australia or New Zealand	700 (63.1)	1091 (66.8)
United Kingdom or Ireland	250 (22.5)	347 (21.2)
Europe	61 (5.5)	82 (5.0)
Asia	56 (5.0)	60 (3.7)
Other	42 (3.8)	53 (3.2)
Highest level of education*		
Less than high school	404 (36.4)	585 (35.8)
Completed high school	223 (20.1)	368 (22.5)
Trade/apprenticeship	241 (21.7)	398 (24.4)
Bachelor degree or higher	241 (21.7)	282 (17.3)
Family history of breast cancer*		
No	668 (60.2)	1170 (71.7)
Yes	438 (39.5)	459 (28.1)
**Number of children**		
None	138 (12.4)	176 (10.8)
1 or more	971 (87.6)	1457 (89.2)
Response*		
One or more causes identified	867 (78.2)	1288 (78.9)
Don’t know	163 (14.7)	131 (8.0)
No response	79 (7.1)	214 (13.1)

*Characteristics that differ significantly (p < 0.05) by Case-Control status

### Risk factors

In the open ended questions (Table 2), case women reported mental/emotional factors most commonly (46%), followed by modifiable/lifestyle factors (39%) and physiological factors (38%). For control women, familial factors were the most commonly reported causes of breast cancer (78%), followed by modifiable/lifestyle (47%) and environmental factors (45%). Case women more often attributed a role in breast cancer causation to chance than control women (Table 2).

**Table 2:** **Risk factor frequencies: BCEES cases (n = 871) and controls (n = 1297) who specified one or more causes**

Risk factors ^a^	Cases N (%)	Controls N (%)
Mental/emotional	404 (46.4)	388 (29.9)
Modifiable/lifestyle	336 (38.6)	611 (47.1)
Physiological	327 (37.5)	291 (22.4)
Environmental	271 (31.1)	589 (45.4)
Genetic	250 (28.7)	1006 (77.6)
Chance	78 (9.0)	84 (6.5)

^a^Note: totals exceed 100% as respondents could identify agents from multiple categories.

### Mental/emotional factors

Almost half of the case women attributed their condition to some aspect of mental state, with a majority specifically citing stress. For many participants, specific stressful life events, including family-life or work-life, were conceptualised as ‘triggers’. Respondents often described a string of events or processes, sometimes extending over many years, that played a causal role in their diagnosis with breast cancer.

*"My inability to deal with a stressful situation over many years resulting in high anxiety and sleeplessness…compounded by a sense of powerlessness over my ability to change the circumstances causing the stress". (Case, 50-54 years)*

*"Excessive stress, anxiety and lack of sleep leading to neglect in diet and lack of exercise and consumption of alcohol – binge drinking". (Case, < 45 years)*

*"Weakening of the immune system caused by stressful events…. making my body lose its natural balance + trigger the cancer". (Case, 55-59 years)*

Control women were less likely than case women to attribute the disorder to mental factors and stress. However, of those control women who did report stress as a casual factor, it was framed in similar terms to the case women.

### Familial factors

The attribution of breast cancer to familial/genetic factors was the most commonly identified cause among control women, a proportion considerably higher than that for case women (Table 2). Control women also reported that the disease could be traced to a ‘biological fault’ which was often considered to lie dormant until a ‘trigger’, such as stress or exposure to pesticides, caused the development of a tumour:

*"…I feel that everyone has a cell that contains cancer but some things trigger that cell to expand, maybe food, chemicals, pesticides or stress". (Control, 60-64 years)*

*"Imbalance in the body cells causes the cancer to become active; hereditary; certain type of genes carrying cancer cells". (Control, 50-54 years)*

### Modifiable/lifestyle factors

A substantial number of participants supplied one or more modifiable or lifestyle factors as breast cancer causes (Table 2). The most frequent attribution by case women in this category was hormone replacement therapy (HRT). Control women, in contrast, were more likely to attribute breast cancer to lifestyle factors such as poor diet, alcohol, smoking, or lack of exercise.

Women from both groups typically listed several different factors from this category:

*"…HRT increases the risk, as does alcohol consumption, obesity, smoking, lack of regular exercise". (Control, 45-49 years)*

*"Lack of physical exercise resulting in weight gain in recent years. Stress; HRT" (Case, 65-69 years)*

Many women also referred to specific foods, both insufficient and in excess. These references were generally combined with a range of other factors:

*"… – alcoholic soft drinks – a diet high in sugar…" (Case, <45 years)*

*"…a lot of red meat, no veges and healthy foods…exercise…" (Control, 50-54 years)*

### Environmental factors

Slightly more control women reported one or more environmental factors as risks for the disease, compared with case women (Table 2). Environmental factors which were mentioned included terms such as ‘chemicals’, ‘pollution’, and ‘toxins’, and alterations to food, such as ‘additives’, ‘preservatives’, ‘pesticides’ and ‘processing’.

*"…the modern environment with chemicals everywhere and in everything, including food, clothing, home, drink (water & manufactured)....polluted air…" (Control, 60-64 years)*

*"…the cumulative effect of pesticides sprayed on the fruit and vegetables and gardens (went into drinking water). Genetically modified food, growth hormones in animal products, stress". (Case, 45-49 years)*

A number of participants also attributed breast cancer development to radiation and more often mentioned non-ionising radiation, such as low frequency magnetic fields, than ionising radiation, such as X-rays and gamma (γ)-radiation:

*"…Also I think factors such as power lines & mobile phone towers play a big part for any type of cancer". (Control, 55-59 years)*

*"…a network computing cabling box sits in that room [work office]....the radiation given off from that electromagnetic field must be enormous". (Case, 50-54 years)*

### Physiological factors

Case women were more likely to report one or more physiological agents as a cause of breast cancer than were control women (Table 2). The most common attributions were reproductive history and hormonal status, both of which are recognised risk factors for breast cancer [26–28]. Respondents also reported physical trauma of the breast as a causal agent.

*"I accidently bumped my breast several times and I believe the result was a small lump that developed into cancer". (Case, 70 years and over)*

## Discussion

Almost 80% of participants provided one or more causes of breast cancer in response to the open-ended question on the RPQ. This finding is consistent with other studies on breast cancer from Australia and Canada [14, 21], and studies of other cancer sites [26, 29] which all confirm that a majority of people with or without a disease have thought about the causes of the disease. In many cases the attribution in this study was tentative, often accompanied by the phrase "I don’t know, but…". The complex aetiology of breast cancer may explain much of this apparent indecision; it is generally acknowledged that interactions between numerous factors and agents are involved in the initiation and development of the disease [27, 30–33].

A large proportion of respondents described more than one causal agent. However, case women gave fewer suggested causes for breast cancer and more often provided a ‘Don’t know’ response, compared with control women. This may reflect the fact that case women were asked what they thought caused their own disease rather than the cause of breast cancer *per se*. Case women will generally have had some discussion with their medical practitioners in an attempt to pinpoint the aetiology of the disease and may have tried to identify a specific cause for their own disease. Previous studies have shown that women with cancer of varying types tend to attribute their disease to fewer agents than they recognise as causal factors for cancer in general [23].

The range of factors cited by respondents was comparable to those reported in previous studies, both those that asked open-ended questions [14, 21] and those that used a checklist format [12, 34]. The most common attribution in control women was familial factors, followed by modifiable/lifestyle and environmental factors, while the most common attribution in case women was mental/emotional factors, followed by modifiable/lifestyle and physiological factors. Two factors included in the Physiological category that are known to increase breast cancer risk, i.e., current age and age at menarche [31, 35], were not commonly cited by respondents.

### Mental/emotional factors

In common with numerous other studies [8, 36, 37] almost half of the case women attributed their own condition to stress or a particular mental state. Similarly, a substantial proportion of the control women associated stress with breast cancer development. The epidemiological evidence does not currently support stress as a risk factor for breast cancer [10, 11], however many women in this study went on to link stress with other factors such as poor diet, lack of exercise and poor sleep. This could imply that, for many women, perceptions of breast cancer development involve a causal chain, with stress forming a vital link in the chain. Another perceived consequence of stress was disruption of the immune system, with a subsequent weakening of protection from developing cancer. As discussed by Wilcox and colleagues [8], there is some evidence for the effect of stress on both lifestyle choices and the immune system, and perhaps the progression of disease, but there is insufficient evidence that stress, either directly or indirectly, causes cancer [8].

### Familial factors

The attribution of breast cancer development to inherited or genetic factors was the most commonly identified cause among control women, a finding previously reported by others [12, 24]. For cases, genetic factors were less commonly mentioned as causes of their cancer. The different reporting of familial factors between the groups is likely to be because the cases would often know that a genetic cause was irrelevant to their own cancer.

While the *BRCA* gene mutations convey a much a higher risk to women who carry them, only a small number of women have the affected genotype, and known inheritable genetic mutations account for less than 5% of breast cancers with other familial risk bringing this to about 9% of breast cancer cases [38, 39]. The perception of breast cancer as an inheritable condition may have implications for public health initiatives on prevention and early detection of the disease if women without a family history of the disease believe that they are not at risk. Education and prevention campaigns targeting breast cancer may need to focus much more strongly on the other identified risk factors for breast cancer, especially those factors that are readily modifiable, and stress the small proportion of breast cancers associated with genetic and familial factors.

### Modifiable/lifestyle factors

The most frequent attribution within the modifiable/lifestyle category by case women was HRT (13.7%). HRT has been identified as a risk factor for breast cancer, and this discovery was the subject of widespread media coverage and policy debate [40, 41]. Given the extensive attention, it was predicted that both case and control women would identify HRT as a risk factor, but this was not the case. The variation in rates of attribution may result from case women having received information from medical professionals that increased their recognition of HRT as a risk factor, or it may represent a reflection of the different question asked of the two groups.

Within this category, control women were more likely to attribute breast cancer to lifestyle factors such as diet, alcohol, smoking, or lack of exercise. Alcohol consumption is a recognised risk factor for breast cancer [30, 42], and moderate and vigorous exercise appears to act as a protective factor [43]. However, there is limited evidence to support a role for smoking or various aspects of diet in the development of the disease [42, 44, 45]. Similarly to reports from the USA [24], respondents identified ‘unhealthy’ factors such as smoking and diet, rather than those that are breast cancer-specific. One study identified that UK adults had limited knowledge of various aspects of lifestyle as risk factors for both cancer and heart disease [46], while a group from the USA also found poor knowledge of breast cancer risk factors [12]. Poor recognition of modifiable lifestyle factors is a significant barrier to effective public health campaigns aimed at disease prevention.

Some lifestyle factors were referred to, by both case and control women, as protective rather than causative: more vegetables and exercise, less fat, red meat and alcohol, and keeping a healthy weight. However, with the exception of low alcohol consumption, sufficient exercise and avoiding weight gain, there is little evidence that specific lifestyle factors protect against breast cancer development [45, 47].

### Environmental factors

Slightly more control women reported one or more environmental factors as risks for breast cancer. However specific chemicals were rarely identified by participants, who generally referred to broad factors such as ‘pollutants’, ‘toxins’ or ‘additives’ in describing environmental risks.

A number of chemicals found in the environment, such as benzene (found in vehicle exhausts), are known carcinogens although with no definite connection to breast cancer [5, 42]. Some pesticides have been shown to mimic the effects of oestrogen and it is plausible that they could increase breast cancer risk, although the current epidemiological evidence is weak [5, 48].

Exposure to ionising radiation, such as x-rays, is a known risk factor for cancers of all types [49], but no role for non-ionising radiation in the development of breast cancer has been established [50, 51]. In this study respondents were more likely to attribute breast cancer to non-ionising than to ionising radiation; this may reflect a poor understanding of the difference between the types of radiation, and the ubiquity of electronic and electrical devices in modern society. Other studies have reported similar attributions of cancer development to both types of radiation [8].

### Strengths of the study

This study used an undirected, open-ended question to elicit the widest possible range of views as to breast cancer causation, similarly to some studies [23], and in contrast to studies based on a checklist of perceived causative factors [12, 19, 22, 24]. In addition, the study had a large sample size of almost 3000 women. The large number of contributions enabled the investigation of nuances, such as the connection of stress with numerous other risk factors, in the causative attributions of women in Western Australia.

### Study limitations

The relatively low response fraction for BCEES may have resulted in an unrepresentative sample. However, the similarity of the findings from this study with previous work across the globe encourages the belief that BCEES participants adequately represent the views of the wider population.

The inclusion of possible risk factors, such as power lines, in the 37-item checklist section of the RPQ which preceded the open-ended question, may have stimulated reporting of these items, to the detriment of other risk factors, such as age and gender, that did not form part of the checklist.

## Conclusion

Overall, many of the risk factors cited by the participants do not correspond to those generally accepted by the scientific community [20]. Some of the divergence may arise from the tendency for negative results to not be published in the scientific literature, and if published, even less likely to be reported in the mass media. Thus one well-publicised study showing a small increase in breast cancer risk due to a particular food, for example, may not be displaced in the minds of the public by poorly-publicised future studies that fail to support the causative role. Second, there may be psychological reasons whereby assigning causation to external factors acts to preserve the individual from self-blame and guilt [37, 52]. In other words, the person believes they have no control over the external exposure and therefore cannot be ‘blamed’ for getting breast cancer.

What is clear however, is that there is a discrepancy between attributed and scientifically-recognised risk factors and that this may have a significant impact on the success of prevention and early detection programs that seek to minimise the pain and suffering caused by this disease. Specifically, the inflated perception of genetic factors as a cause of breast cancer amongst women without breast cancer is a potential threat to the success of prevention and early detection programs. There is a need for research to identify what impact this poor understanding of the role of genetics may be having on mammographic screening, and a role for health promotion in raising awareness of the true role of genetics in breast cancer risk. In addition, the relatively poor recognition of the causative role of some modifiable lifestyle factors is a significant barrier to effective public health campaigns aimed at disease prevention.

There is a need for health promotion campaigns to raise awareness and improve knowledge of the scientifically-recognised risk factors, in order to allow women to make informed choices about their health and healthcare options. There is a role for additional research to quantify the impact of these misperceptions on breast cancer risk and for health promotion to improve knowledge and awareness of risk factors with a strong evidence base.

## Abbreviations

BCEES: Breast cancer, environment & employment study
HRT: Hormone replacement therapy
RPQ: Risk perception questionnaire.
